# The Role of Configurality in the Thatcher Illusion: An ERP Study

**DOI:** 10.3758/s13423-014-0705-3

**Published:** 2014-08-08

**Authors:** Natalie Mestry, Tamaryn Menneer, Michael J. Wenger, Nicholas Benikos, Rosaleen A. McCarthy, Nick Donnelly

**Affiliations:** 1 Psychology, University of Southampton, Southampton, Hampshire SO17 1BJ UK; 2 Psychology, University of Oklahoma, Norman, OK USA; 3 Wessex Neurological Centre, Southampton, UK

**Keywords:** ERP, Thatcher illusion, Configural processing, Face processing, Prosopagnosia

## Abstract

The Thatcher illusion (Thompson in *Perception, 9,* 483–484, 1980) is often explained as resulting from recognising a distortion of configural information when ‘Thatcherised’ faces are upright but not when inverted. However, recent behavioural studies suggest that there is an absence of perceptual configurality in upright Thatcherised faces (Donnelly et al. in *Attention, Perception & Psychophysics, 74,* 1475–1487, 2012) and both perceptual and decisional sources of configurality in behavioural tasks with Thatcherised stimuli (Mestry, Menneer et al. in *Frontiers in Psychology, 3,* 456, 2012). To examine sources linked to the behavioural experience of the illusion, we studied inversion and Thatcherisation of faces (comparing across conditions in which no features, the eyes, the mouth, or both features were Thatcherised) on a set of event-related potential (ERP) components. Effects of inversion were found at the N170, P2 and P3b. Effects of eye condition were restricted to the N170 generated in the right hemisphere. Critically, an interaction of orientation and eye Thatcherisation was found for the P3b amplitude. Results from an individual with acquired prosopagnosia who can discriminate Thatcherised from typical faces but cannot categorise them or perceive the illusion (Mestry, Donnelly et al. in *Neuropsychologia, 50,* 3410-3418, 2012) only differed from typical participants at the P3b component. Findings suggest the P3b links most directly to the experience of the illusion. Overall, the study showed evidence consistent with both perceptual and decisional sources and the need to consider both in relation to configurality.

**Electronic supplementary material:**

The online version of this article (doi:10.3758/s13423-014-0705-3) contains supplementary material, which is available to authorized users.

The Thatcher illusion (Thompson, 1980) describes how the grotesque appearance of an upright ‘Thatcherised’ face, where the eyes and mouth are inverted relative to the face context, disappears when the face is rotated from upright. The Thatcher illusion is used as evidence to support the dual-mode hypothesis of face processing (e.g. Bartlett & Searcy, 1993), that configural information is utilised in upright faces, but is less accessible in inverted faces whilst feature information is relatively unaffected by orientation. The dual-mode account explains the illusion as experiencing the unusual configural relations in upright but not inverted Thatcherised faces.

The role of configural processing in the Thatcher illusion has been explored in two previous studies (Cornes, Donnelly, Godwin & Wenger, 2011; Mestry, Menneer, Wenger & Donnelly, 2012), conducted using the framework of general recognition theory (GRT; Ashby & Townsend, 1986). In these tasks, participants made separate judgements to the orientation of eyes and mouths where the features were manipulated in a complete factorial design. Faces were typical or had the eyes, the mouth, or both features Thatcherised. Responses to eyes and mouths defined four bivariate response distributions. Using multidimensional signal detection analyses, sensitivity and bias were calculated and compared across pairs of distributions to test for three variants of configurality that are theoretically (and mathematically) defined within GRT. Both Cornes et al. (2011) and Mestry, Menneer et al. (2012) found evidence of interdependencies between the discrimination of feature orientation of eyes and mouths at both perceptual and decisional levels.

Mestry, Menneer et al. (2012) further investigated one particular form of perceptual configurality: violation of perceptual independence. This measure defines configurality as a correlation between perception of the eyes and mouth in a single face. By estimating the bivariate correlations of each of the individual response distributions using probit analysis (DeCarlo, 2003), significant correlations between eyes and mouths were shown to exist, but only if at least one feature was upright. Critically, if both eyes and mouths were inverted in an upright face (as in the Thatcher illusion), then there was no correlation between eyes and mouths. These data suggest that upright typical faces are subject to this form of perceptual configural processing, but that upright Thatcherised faces are not.

The same conclusion about the absence of configural processing in upright Thatcherised faces emerges from a third study (Donnelly, Cornes & Menneer, 2012). This study used systems factorial technology (see Townsend & Nozawa, 1995) to analyse response time distributions to faces. Response time distributions to decisions about typicality versus Thatcherisation using typical, partially Thatcherised and fully Thatcherised faces were compared. The goal was to determine if Thatcherised faces are categorised from typical faces in a system using super, unlimited or limited capacity. Configural processing must, by definition, be associated with systems operating in supercapacity. Supercapacity means that the configuration speeds responses such that they are faster than can be predicted from responses to the elements forming the configuration (Townsend & Nozawa, 1995). In the study, detection of Thatcherisation was associated with unlimited and limited capacity processing, but almost never with supercapacity processing (Donnelly et al., 2012).

Together, the findings from Cornes et al. (2011), Mestry, Menneer et al. (2012) and Donnelly et al. (2012) suggest two qualifications to the standard account of how Thatcherised faces are processed relative to typical faces. First, upright Thatcherised faces are not subject to perceptual configural processing. Second, the processing of Thatcherised faces differs from that of typical faces in ways that are subject to influence from perceptual and decisional factors. Both findings challenge the dual-mode account of the Thatcher illusion. In the present study, we seek converging evidence from event-related potentials (ERPs) for perceptual and decisional influences in a task with Thatcherised faces.

Previous ERP studies of the Thatcher illusion have focussed on perceptual markers (P1, N170 and P2). Therefore, the possibility that the Thatcher illusion is contributed to by decisional influences in ERP components has not previously been explored. With respect to perceptual markers of the Thatcher illusion, Thatcherised faces (compared to typical faces) have been shown to lead to reduced (Boutsen, Humphreys, Praamstra, & Warbrick, 2006) and increased (Milivojevic, Clapp, Johnson, & Corballis, 2003; Carbon, Schweinberger, Kaufmann, & Leder, 2005) amplitude of the N170. Both Milivojevic et al. (2003) and Carbon et al. (2005) report interactions of Thatcherisation and orientation on amplitude of N170. However, there was no consistency in the form of the interaction. Although Milivojevic et al. (2003) explored a longer epoch and found differences at the parietal component (450–600 ms), they suggested these findings were associated with featural processing. Furthermore, all previous ERP studies have compared Thatcherised to typical faces without considering partially Thatcherised faces. This means that the critical stimulus conditions that underlie any particular stimulus effect cannot be resolved beyond a simple comparison of typical and fully Thatcherised faces.

To resolve these issues, the present study examines upright and inverted faces with both features Thatcherised, eyes or mouth Thatcherised, or no features Thatcherised in a sample of participants with typical face processing. We also explored these effects in an epoch large enough to capture the P3b, a later component suggested to reflect a decisional ‘monitoring’ process (Verleger, Jaśkowski, & Wascher, 2005). We predicted that, due to evidence reporting both perceptual and decisional forms of configurality in previous tasks exploring the Thatcher illusion, effects throughout the information processing chain at early and late components reflecting both perceptual and decisional configurality may be found.

We also tested PHD, an individual with acquired prosopagnosia. PHD has no N170 face effect (Eimer & McCarthy, 1999) and is unable to categorise individual faces as Thatcherised or typical, but is able to make same/different judgements to simultaneously presented pairs of Thatcherised and typical faces (Mestry, Donnelly, Menneer & McCarthy, 2012). We tested PHD to explore which markers present in the ERP traces of typical participants would disappear for PHD in the absence of sensitivity to the illusion. This was in order to identify those ERP markers associated with being able to discriminate Thatcherised from typical faces but not with experiencing the illusion itself.

## Method

### Participants

Twenty-three participants with normal or corrected-to-normal vision were recruited via an opportunity sample and participated in return for payment. Ages ranged from 19 to 43 (*M* = 25.52, *SD* = 5.25) and eight participants were male. A 52-year-old male with acquired prosopagnosia (PHD) was also recruited to take part in the study. All participants were non-smokers, with no recent history of epilepsy or the use of psychoactive medication.

### Design

A two (orientation: upright or inverted face) × two (eye condition: normal or Thatcherised) × two (mouth condition: normal or Thatcherised) repeated measures design was used. Each participant completed 240 trials in each of the eight conditions, randomised across 16 blocks.

### Stimuli

**Fig. 1:**
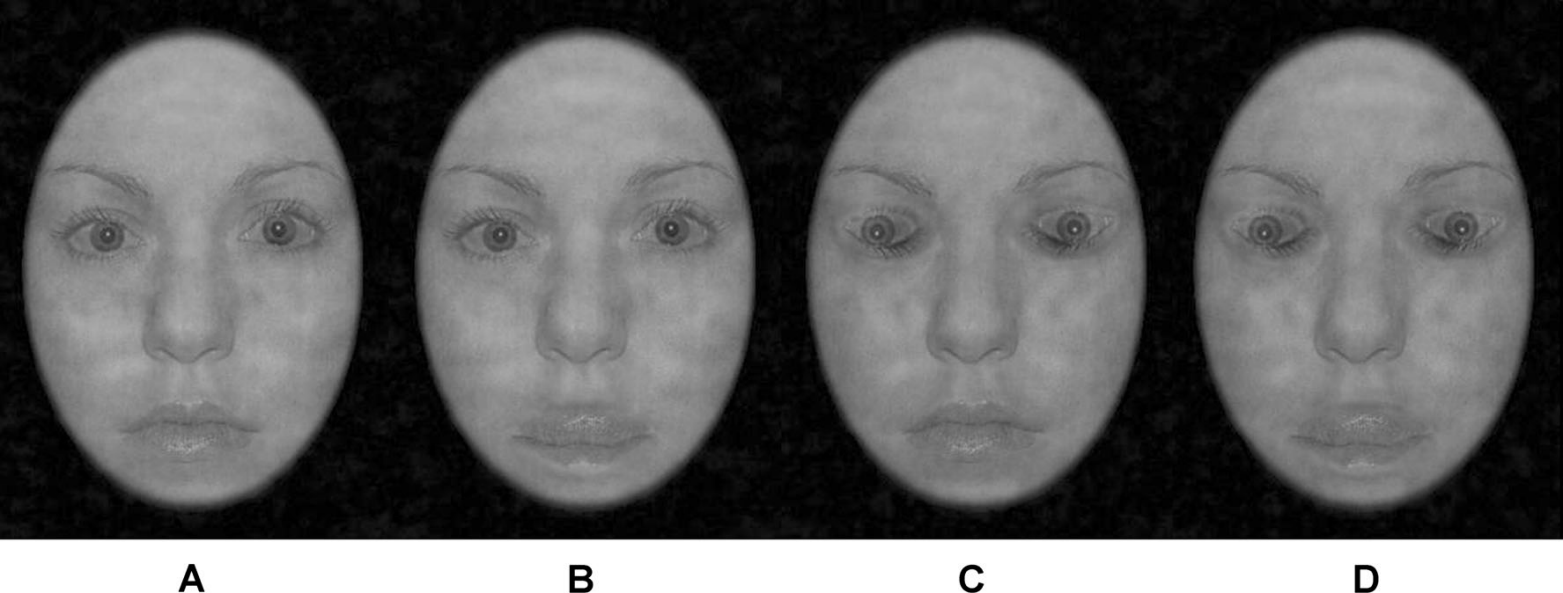
Face stimuli used in the experiment. Thatcherisation conditions: **a **both eyes and mouth in the normal orientation; **b **eyes normal, mouth Thatcherised; **c** eyes Thatcherised, mouth normal; and **d **eyes and mouth Thatcherised

Forty grey-scale faces from the set created by Mestry, Menneer et al. (2012), derived from a subset of the NimStim face stimuli (Tottenham et al., 2009), served as base stimuli. These images represented ten individuals across the four manipulation conditions (Fig. 1) and were also inverted to create 80 faces in total. A two-dimensional fast Fourier transform was applied to equate spatial frequency and a Butterworth filter was used to remove the influence of the face outline (Rousselet, Husk, Bennett, & Sekuler, 2008). Faces were presented in the centre of the screen at a visual angle of 3.44° × 4.80° when viewed from a distance of 100 cm.

### Apparatus and Materials

All stimuli were presented in a darkened room on a desktop computer with a screen size of 32.80 cm × 24.50 cm. Screen resolution was 1024 × 768, and with a refresh rate of 85 Hz. Participants responded by clicking buttons on a response button-box. All stimuli were presented against a black background and text instructions were presented in white. EEG was recorded using SCAN 4.4™ (© 2006, Compumedics Neuroscan).

### Procedure

Participants made speeded judgements of face orientation (upright or inverted). This task was chosen to avoid difficulties performing the task that could lead to differences at ERP components (e.g. Milivojevic et al. (2003) found differences at P3 due to difficulty discriminating gender), but also to promote a global strategy and reduce feature-based processing. On each trial, a blank black screen was presented for 200 ms followed by a small white fixation cross in the centre of the screen for a random duration ranging from 500 to 900 ms. A stimulus was then presented for 100 ms, followed by a blank screen for 1000 ms during which time a response was required. If no response was made, then this trial was treated as incorrect. Participants were offered breaks between blocks.

### ERP

**Fig. 2:**
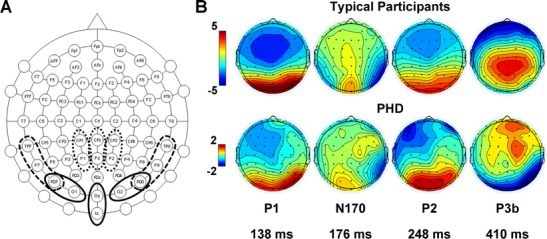
**a** Locations of all electrodes used (those named only). Clusters of electrodes used for analysis of each component are indicated by the *bold lines*: *solid line* for P1 and P2, *dashed line* for N170 and *small dotted line* for P3b. The active electrode was placed at AF_z_ and ground at FC_z_. The VEOG was monitored by placing electrodes above and below the right eye. **b** Topographical distributions for typical participants and PHD for each of the P1, N170, P2 and P3b components, with time (ms) representing the peak latency at the centre of the peak detection window for that component

EEG data were acquired from 60 electrodes (Fig. 2) using a 10–20 system Easycap (Brain Products) and a SynAmps^2^ amplifier headbox (Compumedics Neuroscan) using Ag/Ag Cl electrodes. The analogue signal was digitalised at 500 Hz and band-pass filtered between 0.1 and 100 Hz. Impedances were kept below 10 kΩ and typically below 5 kΩ. Participants were asked to minimise movement.

The EEG data were re-referenced offline to an average reference. Electrodes were rejected on a participant-by-participant basis. The signal was low-pass filtered at 30 Hz (48 dB/octave). Baseline correction was performed using 300 ms of pre-stimulus activity. Eye movements were corrected using an ocular artefact reduction algorithm for the VEOG channel (Semlitsch, Anderer, Schuster, & Presslich, 1986). Artefacts were rejected based on absolute values larger than 100 μV (50 μV for PHD). Only correct trials were averaged using an interval from –300 ms to +800 ms.

Sixteen participants were included in the final sample. Two were excluded for low task accuracy (<80 %), one for poor impedance values (>10 kΩ) and four because fewer than 40 trials per condition remained after pre-processing. Across the participants, the number of trials per condition ranged between 40 and 208 (*M* = 126.56) out of 240 possible trials. There were no differences among conditions for the number of trials contributed by each participant (*F*_7,105_ = 1.18, *p* = 0.322). For PHD, the number of trials per condition ranged between 64 and 84 (*M* = 71.13).

Global Field Potential (GFP; Lehmann & Skrandies, 1980) was calculated for each participant in each of the eight conditions. The P1, N170, P2 and P3b components emerged from the grand average for all typical participants across the task (Supplementary Fig. 1). Component windows for peak detection were defined from the electrode of maximum amplitude for each component (Picton et al., 2000) with 10 samples either side of the peak for the P1, N170 and P2, and 50 samples either side of the peak for the P3b to estimate the top third of the peak. Electrodes for peak detection were selected based on electrodes of maximum amplitude in the grand average GFP for each of the component windows and the corresponding electrodes from the opposite hemisphere, until six electrodes were selected for each component, these electrodes were then analysed as clusters (Fig. 2).

## Results

### Behavioural Results

Average accuracy and correct response times (RTs) across the conditions are provided in Supplementary Table 1. A repeated measures factorial ANOVA of orientation (upright and inverted), eye condition (normal or Thatcherised) and mouth condition (normal or Thatcherised) was conducted on mean RTs from correct trials in each condition. The main effect of orientation was significant (*F*_1,15_ = 8.00, *p* = 0.013, *η*_*p*_^2^ = 0.348). RTs were faster to upright (*M* = 613.53, *SE* = 19.92) than inverted (*M* = 633.14, *SE* = 23.89) faces. The main effect of mouth condition was significant (*F*_1,15_ = 5.98, *p* = 0.027, *η*_*p*_^2^ = 0.285). RTs were faster to normal (*M* = 620.34, *SE* = 21.43) than Thatcherised (*M* = 626.33, *SE* = 22.08) mouths. Neither the main effect of eye condition nor the interactions reached significance (*Fs*_1, 15_ < 2.55, *ps* > 0.131).

### ERP Results

Peak amplitude and latency (time to peak amplitude) were compared using repeated measures ANOVAs for each component (Supplementary Table 2). Mean values were computed for each electrode cluster (see Milivojevic et al., 2003). A three (hemisphere: left (LH), midline and right (RH)) × two (orientation: upright or inverted) × two (eyes: normal or Thatcherised) × two (mouth: normal or Thatcherised) design was used for each component except for the N170, where hemisphere only had two levels (LH and RH). Condition means for significant effects are reported in Table 1. Pairwise comparisons with Bonferroni correction were used to identify reliable differences between levels for the main effects, and separate ANOVAs were used to interpret the interactions.

**Table 1:** Condition means for significant ANOVA effects computed for peak amplitude and latency of each component

Variable	Amplitude (μV)		Latency (ms)	
	*M*	*SE*	*M*	*SE*
P1				
Orientation × Mouth				
Inverted				
Normal	-	-	137.67	1.86
Thatcherised	-	-	139.03	1.82
Upright				
Normal	-	-	137.85	1.90
Thatcherised	-	-	137.98	1.80
N170				
Orientation				
Inverted	–4.41	0.82	190.37	1.99
Upright	–3.84	0.75	185.77	2.06
Hemisphere × Eye Condition				
Left				
Normal	–3.15	0.54	-	-
Thatcherised	–3.35	0.58	-	-
Right				
Normal	–5.11	1.16	-	-
Thatcherised	–4.90	1.12	-	-
P2				
Hemisphere				
Left	-	-	248.32	2.47
Midline	-	-	246.32	2.31
Right	-	-	244.83	2.11
Orientation				
Inverted	3.89	0.68	-	-
Upright	4.69	0.58	-	-
Hemisphere × Orientation × Eye condition				
Left				
Inverted				
Normal	-	-	249.09	2.90
Thatcherised	-	-	249.22	2.66
Upright				
Normal	-	-	247.09	2.48
Thatcherised	-	-	247.88	2.48
Midline				
Inverted				
Normal	-	-	246.81	2.56
Thatcherised	-	-	248.59	2.31
Upright				
Normal	-	-	245.19	2.47
Thatcherised	-	-	244.69	2.33
Right				
Inverted				
Normal	-	-	243.81	2.26
Thatcherised	-	-	245.66	2.27
Upright				
Normal	-	-	245.44	2.30
Thatcherised	-	-	244.41	2.33
P3b				
Hemisphere				
Left	4.49	0.32	-	-
Midline	5.24	0.39	-	-
Right	4.72	0.46	-	-
Orientation				
Inverted	5.13	0.43	-	-
Upright	4.50	0.34	-	-
Hemisphere × Orientation × Eye condition				
Left				
Inverted				
Normal	4.80	0.37	409.53	10.02
Thatcherised	4.81	0.40	408.44	9.42
Upright				
Normal	4.18	0.31	411.56	11.54
Thatcherised	4.16	0.28	405.50	13.76
Midline				
Inverted				
Normal	5.57	0.43	410.09	10.71
Thatcherised	5.72	0.46	412.06	11.67
Upright				
Normal	4.82	0.37	415.03	12.22
Thatcherised	4.84	0.35	405.69	13.67
Right				
Inverted				
Normal	4.83	0.50	411.97	9.70
Thatcherised	5.05	0.53	409.47	11.20
Upright				
Normal	4.56	0.45	405.16	12.95
Thatcherised	4.44	0.44	410.13	13.23

Data from typical participants. Means from significant ANOVA effects only. For significant interactions of latency, if the source of the interaction was a difference in latency below the sampling rate of 2 ms, they will not be considered further. These included the interaction of orientation and mouth condition for P1 and the interactions of hemisphere, orientation and eyes for P2 and P3b

**Fig. 3:**
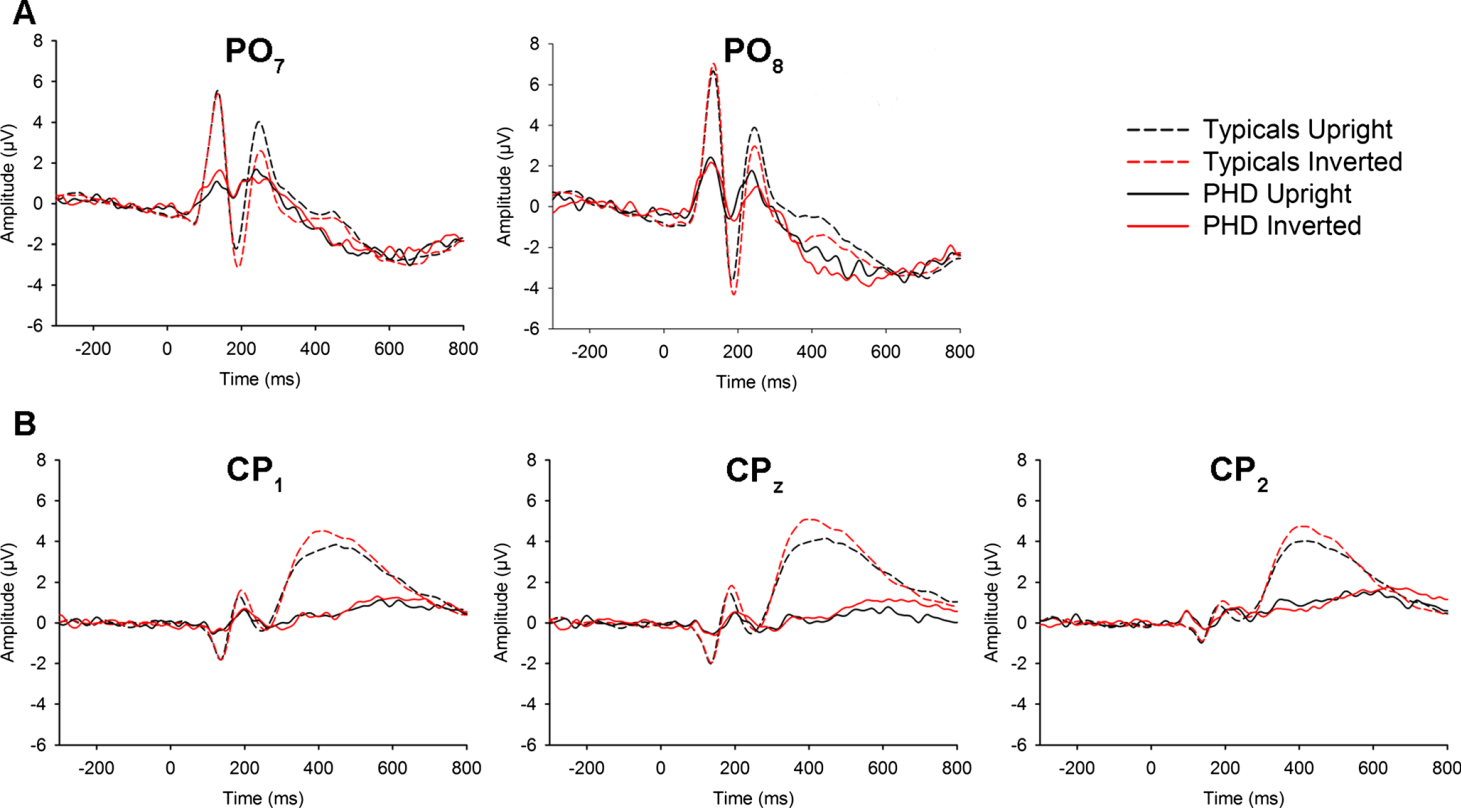
Amplitude (μV) across upright and inverted face condition. **a** PO_7_ and PO_8_ electrodes providing examples of inversion effects for the N170, P2 for typical participants and PHD; **b** CP_1_, CP_z_ and CP_1_ electrodes providing examples of inversion effects for the P3b for typical participants and not PHD

The N170 was larger and emerged later for inverted than upright faces (Fig. 3). Eye condition was significant for the RH but not LH N170 (*F*_1,15_ = 7.73, *p* = 0.014, *η*_*p*_^2^ = 0.34; *F*_3,45_ = 3.39, *p* = 0.085, *η*_*p*_^2^ = 0.18. respectively). Normal eyes led to a larger N170 than Thatcherised eyes in the RH but not the LH (see Table 1).

The P2 was larger for upright than inverted faces (Fig. 3). For latency, pairwise comparison revealed no significant differences between the different electrode clusters across hemisphere, but there was a significant linear trend (*F*_1,15_ = 6.34, *p* = 0.024, *η*_*p*_^2^ = 0.30). Time to peak amplitude was shorter in the RH cluster, followed by the midline and LH clusters.

With respect to hemisphere, pairwise comparison revealed significant differences between the P3b amplitudes at the midline and LH electrode clusters (*p* = 0.001), midline and the RH electrode clusters (*p* = 0.009) but not between the RH and LH clusters (*p* = 1.000). With respect to orientation, peak P3b amplitude was larger for inverted than upright faces (Fig. 3). The interaction of hemisphere, orientation and eye condition was significant for P3b. There was no difference in amplitude to normal and Thatcherised eyes across orientation in the LH. At the midline and in the RH, amplitude was increased to Thatcherised relative to normal eyes for inverted faces. In the RH, Thatcherised eyes also led to reduced amplitude relative to normal eyes for upright faces forming a crossover interaction (see Table 1).

### Correlations between ERP and RT

In order to determine the extent to which measured brain activity related to behavioural responses, we calculated pair-wise correlations between the peak amplitude and latency of the four ERP components and the correct RTs for judgements of stimulus orientation (see Supplementary Table 3). There was a significant negative correlation between N170 amplitude and RT, meaning increased RT was associated with the more negative amplitude values (the larger deflections).

### Prosopagnosia

Accuracy and correct RTs across for PHD are presented in Supplementary Table 1. Like typicals, PHD was faster classifying upright than inverted faces.

All four ERP components were present in a plot of PHD’s GFP (Supplementary Fig. 1). Peak amplitude and latency for each of the components were selected from PHD’s data using the parameters outlined for typical participants. Only the effects significant in the typical data were explored in PHD’s ERP to see if they remained in absence of sensitivity to experience the illusion.

First, PHD showed evidence of significant inversion effects at N170, P2 and P3b. We calculated difference scores (inverted – upright) and computed adjusted one-sample *t* tests appropriate for a modest control sample (Crawford & Howell, 1998) to compare PHD to typical participants (Table 2). There were no differences in the sizes of the inversion effects for typical participants and PHD. Given differences in the absolute magnitude of ERP components for typicals and PHD, we also computed confidence intervals on the average amplitude difference scores for the component electrode cluster before stimulus onset for PHD. If PHD’s difference value fell outside of this range, then we deemed the inversion effect unreliable (see Supplementary Table 4). The size of these inversion effects for PHD at N170 and P2 were found to be reliable but not at the P3b (Fig. 3).

**Table 2:** One-sample *t* test results comparing size of inversion effect and eye Thatcherisation effect for PHD to typical participants

Variable	Typical participants		Test value (PHD)	*t*	*p*
	*M*	*SD*			
Inversion effect					
N170					
Amplitude	–0.571	0.797	–0.087	0.589	0.565
Latency	4.604	3.509	1.833	–0.766	0.456
P2					
Amplitude	–0.797	0.974	0.473	1.265	0.225
P3b					
Amplitude	0.631	0.725	–0.010	–0.858	0.405
Eye Thatcherisation effect					
N170					
Amplitude	–0.218	.313	–.074	0.446	0.662

*n* = 16. Values represent difference scores (inverted – upright for inversion effect and normal – Thatcherised for eye Thatcherisation effect). Bonferroni correction for multiple comparisons (*p* = 0.05/5 = 0.01). We will not consider PHD’s N170 latency effect further as the difference in latency was below the sampling rate of 2 ms

We also examined effect of eye condition at N170 in the RH by computing difference scores (normal – Thatcherised eye condition). PHD showed reduced amplitude to Thatcherised eyes relative to normal eyes (Table 2) that was reliably different from baseline (Supplementary Table 4; Supplementary Fig. 2). However, the eye condition and orientation interaction at P3b found at the midline and RH in typicals was not present for PHD (Supplementary Fig. 3).

## Discussion

The present study explored ERP responses to typical, partially Thatcherised and fully Thatcherised faces while participants made judgements of the orientation of faces. Our study provides several novel findings that improve understanding of the role of configurality in the Thatcher illusion.

Inversion of faces led to significant differences at the N170, P2 and P3b components. The N170 and P2 components reflect perceptual processes (Boutsen et al., 2006) and the P3b reflects decisional processes (Verleger et al., 2005). Therefore, a new finding is that both perceptual and decisional processing are used in the task.

The second new finding is the influence of eyes in determining the amplitude of the N170. The reduced amplitude of N170 for Thatcherised versus normal eyes in faces in the RH replicates the effect of Thatcherisation reported by Boutsen et al. (2006). The effect with eyes is consistent with functional imaging data reported previously (Donnelly et al., 2011). Larger N170 amplitudes were associated with faster RTs to judgements of overall face orientation. The simplest interpretation of this relationship between amplitude of the N170 and RT is that the amplitude is reduced when information about eye orientation interferes with that determining overall face orientation.

The third new finding we report is that only at the P3b did face orientation and Thatcherisation interact, and then only for eyes. ERPs at the P3b are influenced by the overall orientation of faces and the orientation of eyes. Normal eyes (relative to Thatcherised eyes) increase ERP amplitude in upright faces but reduce it in inverted faces. Thatcherised eyes in inverted faces are upright when considered in a viewer-centred rather than facial reference frame. Eyes that are upright, regardless of the facial frame, give rise to larger P3b than inverted eyes. This simple observation leads us to conclude that the modulating effect of eyes on the effect of face orientation on P3b occurs through eye orientation also being coded in a viewer-centred rather than facial reference frame. The role of different reference frames in the Thatcher illusion have been considered previously (Parks, 1983; Valentine & Bruce, 1985) and we suggest that, when Thatcherised eyes are presented in upright faces, resolution of the two signals (face upright, eyes inverted) leads the sense of grotesqueness.

Together these data lead to an important conclusion. The Thatcher illusion has two potential sources. One source is perceptual (N170) and the other is decisional (P3b). Determining how either component is expressed in terms of the phenomenology of the illusion is beyond the scope of the present study. To do so would require an understanding of how modulations of amplitude in ERP map on to phenomenological experience and we have no such understanding. Nevertheless, the data from PHD do allow us to make some comment.

PHD showed the modulation of the N170 by eye orientation at the N170. While this component may be critical to discriminating Thatcherised from typical faces, it does not lead to any experience of grotesqueness in PHD. We suggest, therefore, that it may not do so in typical participants.

PHD showed very different results from typicals at the P3b. From PHD’s GFP, we can see that the component appears to peak much later. None of the ERP markers found for typical individuals at P3b emerge; there is no inversion effect at the P3b time window and no pattern of results matching the hemisphere by orientation by eye interaction. While failure to find markers may be in part due to PHD’s overall attenuation of amplitude and baseline noise in the ERP, his P3b component is different to typical participants. Therefore, the P3b is the one component that seems likely to link to our experience of grotesqueness with respect to the Thatcher illusion. PHD’s data have been key in this understanding.

The results challenge the idea that the experience of the Thatcher illusion corresponds to a modulation of the N170. In doing so, the data challenge accounts of the illusion constructed in terms of perceptual relational processing. Instead, the data show a role for decisional processing, indexed by the P3b.

In conclusion, the N170 appears important to discriminating Thatcherised from typical faces, but it is the P3b that appears to link to the experience of the illusion. The role of configurality in the illusion may have a decisional source. Therefore, we suggest the need to consider both perceptual and decisional contributions to configurality.

## Electronic supplementary materials

Below is the link to the electronic supplementary material.

Supplementary Table 1 (PDF 27 kb)

Supplementary Table 2 (PDF 70 kb)

Supplementary Table 3 (PDF 25 kb)

Supplementary Table 4 (PDF 25 kb)

Supplementary Fig. 1 (PDF 223 kb)

Supplementary Fig. 2 (PDF 198 kb)

Supplementary Fig. 3 (PDF 145 kb)
