# Supplementary material for: The Role of Configurality in the Thatcher Illusion: An ERP Study
Source: Psychon Bull Rev. 2014 Aug 8;22(2):445–52. doi: 10.3758/s13423-014-0705-3 (PMC4365276; doi:10.3758/s13423-014-0705-3)
Supplement: Supplementary file 1 — (PDF 27 kb) [file 13423_2014_705_MOESM1_ESM.pdf]

Supplementary Table 1

Mean Accuracy and Mean Correct Response Times across the Upright and Inverted Face Conditions

	Upright				Inverted			
	Eyes Normal		Eyes Thatcherised		Eyes Normal		Eyes Thatcherised	
Variable	N	T	N	T	N	T	N	T
Typical Participants								
Accuracy	96.12	94.77	95.70	94.11	94.51	94.06	94.87	93.46
(%)	(2.14)	(2.32)	(2.51)	(3.56)	(2.41)	(3.46)	(2.72)	(2.87)
Response	601.44	610.49	604.55	611.51	625.21	629.68	624.89	627.19
Time (ms)	(80.23)	(83.48)	(83.29)	(88.02)	(94.27)	(98.68)	(93.65)	(94.46)
PHD								
Accuracy								
(%)	90.00	92.08	93.33	90.00	93.33	89.58	93.75	92.08
Response	845.03	811.26	804.20	830.43	839.41	832.63	839.21	881.48
Time (ms)	(252.87)	(176.99)	(196.37)	(214.29)	(189.13)	(187.69)	(220.24)	(266.15)

Note. *SD* presented in brackets. N = 16 for typical participants. N = normal, T = Thatcherised for mouth condition.

For Review Only
