# Supplementary material for: The Role of Configurality in the Thatcher Illusion: An ERP Study
Source: Psychon Bull Rev. 2014 Aug 8;22(2):445–52. doi: 10.3758/s13423-014-0705-3 (PMC4365276; doi:10.3758/s13423-014-0705-3)
Supplement: Supplementary file 2 — (PDF 70 kb) [file 13423_2014_705_MOESM2_ESM.pdf]

Supplementary Table 2

Summary of ANOVA Results for Peak Amplitude and Peak Latency Data from Each Component

	Amplitude (μV)				Latency (ms)			
Variable	*df*	*F*	*p*	η_p^2	*df*	*F*	*p*	η_p^2
P1								
H	2, 30	2.19	0.130	0.127	2, 30	2.78	0.078	0.156
O	1, 15	2.19	0.159	0.128	1, 15	0.59	0.453	0.038
E	1, 15	3.13	0.097	0.173	1, 15	0.03	0.874	0.002
M	1, 15	0.13	0.727	0.008	1, 15	1.33	0.266	0.082
H x O	1.20, 18.02	1.42	0.255	0.087	1.08, 16.26	1.17	0.300	0.072
H x E	2, 30	0.75	0.479	0.048	2, 30	1.80	0.182	0.107
O x E	1, 15	3.04	0.102	0.168	1, 15	0.21	0.654	0.014
H x O x E	2, 30	1.56	0.226	0.094	1.29, 19.31	0.38	0.600	0.024
H x M	2, 30	2.21	0.128	0.128	1.45, 21.77	1.76	0.200	0.105
O x M	1, 15	0.49	0.495	0.032	**1, 15**	**4.69**	**0.047**	**0.238**
H x O x M	1.17, 17.56	0.19	0.709	0.012	2, 30	1.35	0.275	0.082
E x M	1, 15	0.13	0.724	0.009	1, 15	0.76	0.397	0.048
H x E x M	2, 30	2.09	0.141	0.122	1.31, 19.57	0.26	0.677	0.017
O x E x M	1, 15	0.02	0.883	0.001	1, 15	0.04	0.839	0.003
H x O x E x M	2, 30	0.02	0.985	0.001	2, 30	0.54	0.589	0.035
N170								
H	1, 15	3.90	0.067	0.206	1, 15	1.56	0.231	0.094
O	**1, 15**	**8.22**	**0.012**	**0.354**	**1, 15**	**27.55**	**0.000**	**0.647**
E	1, 15	0.02	0.894	0.001	1, 15	0.00	0.979	0.000
M	1, 15	2.64	0.125	0.150	1, 15	0.23	0.637	0.015
H x O	1, 15	0.40	0.536	0.026	1, 15	1.71	0.210	0.102
H x E	**1, 15**	**14.68**	**0.002**	**0.495**	1, 15	3.46	0.083	0.187
O x E	1, 15	3.98	0.065	0.210	1, 15	2.40	0.142	0.138

H x O x E	1, 15	1.21	0.289	0.075	1, 15	0.01	0.930	0.001
H x M	1, 15	1.25	0.280	0.077	1, 15	0.20	0.659	0.013
O x M	1, 15	2.05	0.173	0.120	1, 15	2.05	0.173	0.120
H x O x M	1, 15	2.43	0.140	0.139	1, 15	3.85	0.069	0.204
E x M	1, 15	0.11	0.740	0.008	1, 15	0.47	0.505	0.030
H x E x M	1, 15	0.83	0.376	0.052	1, 15	0.74	0.403	0.047
O x E x M	1, 15	0.00	0.970	0.000	1, 15	0.71	0.414	0.045
H x O x E x M	1, 15	0.02	0.895	0.001	1, 15	0.46	0.507	0.030
P2								
H	2, 30	0.49	0.616	0.032	**2, 30**	**4.20**	**0.025**	**0.219**
O	**1, 15**	**10.73**	**0.005**	**0.417**	1, 15	1.80	0.200	0.107
E	1, 15	2.08	0.170	0.122	1, 15	0.62	0.444	0.040
M	1, 15	0.00	0.998	0.000	1, 15	0.02	0.894	0.001
H x O	1.13, 16.87	2.61	0.122	0.148	1.39, 20.80	2.51	0.120	0.143
H x E	1.42, 21.35	0.44	0.585	0.028	1.38, 20.74	0.05	0.903	0.003
O x E	1,	2.64	0.125	0.149	1, 15	1.43	0.250	0.087
H x O x E	1.17, 17.58	0.49	0.522	0.032	**2, 30**	**4.05**	**0.028**	**0.213**
H x M	2,	1.84	0.177	0.109	2, 30	0.64	0.533	0.041
O x M	1,	0.86	0.370	0.054	1, 15	0.05	0.836	0.003
H x O x M	1.47, 21.98	2.33	0.132	0.135	1.45, 21.68	0.41	0.603	0.027
E x M	1,	1.50	0.239	0.091	1, 15	0.03	0.870	0.002
H x E x M	2,	2.85	0.074	0.159	1.20, 17.94	0.06	0.847	0.004
O x E x M	1,	0.10	0.751	0.007	1, 15	3.52	0.080	0.190
H x O x E x M	1.46, 21.82	1.52	0.240	0.092	1.38, 20.75	0.02	0.950	0.001
P3b								
H	**1.20, 18.05**	**7.42**	**0.011**	**0.331**	1.39, 20.87	0.17	0.762	0.011
O	**1, 15**	**12.09**	**0.003**	**0.446**	1, 15	0.04	0.845	0.003
E	1, 15	0.21	0.657	0.013	1, 15	0.27	0.612	0.018
M	1, 15	0.48	0.497	0.031	1, 15	0.05	0.830	0.003
H x O	1.26, 18.82	2.04	0.168	0.120	1.30, 19.47	0.09	0.833	0.006

H x E	2, 30	1.33	0.280	0.081	2, 30	0.99	0.383	0.062
O x E	1, 15	0.68	0.424	0.043	1, 15	0.27	0.612	0.018
H x O x E	**2, 30**	**3.35**	**0.049**	**0.183**	**2, 30**	**4.19**	**0.025**	**0.218**
H x M	1.33, 19.94	2.19	0.150	0.127	2, 30	0.68	0.514	0.043
O x M	1, 15	0.00	0.949	0.000	1, 15	0.00	0.996	0.000
H x O x M	1.36, 20.40	1.42	0.257	0.087	2, 30	0.41	0.667	0.027
E x M	1, 15	0.00	0.994	0.000	1, 15	0.01	0.937	0.000
H x E x M	1.29, 19.35	0.85	0.396	0.054	2, 30	1.41	0.260	0.086
O x E x M	1, 15	0.09	0.775	0.006	1, 15	0.05	0.835	0.003
H x O x E x M	2, 30	0.16	0.856	0.010	2, 30	0.22	0.806	0.014

Note. Data from Control Participants only. Significant results at $p < 0.05$ highlighted in bold. Greenhouse-Geisser correction reported if there was a violation of Mauchly's test of sphericity. Notation denotes: hemisphere (H), orientation (O), eye condition (E), mouth condition (M).

For Review Only
