# Supplementary material for: The Role of Configurality in the Thatcher Illusion: An ERP Study
Source: Psychon Bull Rev. 2014 Aug 8;22(2):445–52. doi: 10.3758/s13423-014-0705-3 (PMC4365276; doi:10.3758/s13423-014-0705-3)
Supplement: Supplementary file 3 — (PDF 25 kb) [file 13423_2014_705_MOESM3_ESM.pdf]

Supplementary Table 3

*Correlations between Correct RTs and Peak Amplitude and Latency for each ERP Component*

| | Amplitude | | Latency | |
|---|---|---|---|---|
| Component | *r* | *p* | *r* | *p* |
| P1 | -0.051 | 0.564 | 0.068 | 0.447 |
| N170 | -0.379 | 0.000* | -0.156 | 0.078 |
| P2 | 0.089 | 0.316 | -0.023 | 0.798 |
| P3b | 0.203 | 0.021 | 0.238 | 0.007 |

*Note.* Data from typical participants only. N = 128 and * indicates significance at $p < 0.05$ using Bonferroni adjusted alpha levels of 0.006 per test ($p = 0.05/8$).

For Review Only
