# Supplementary material for: The Role of Configurality in the Thatcher Illusion: An ERP Study
Source: Psychon Bull Rev. 2014 Aug 8;22(2):445–52. doi: 10.3758/s13423-014-0705-3 (PMC4365276; doi:10.3758/s13423-014-0705-3)
Supplement: Supplementary file 4 — (PDF 25 kb) [file 13423_2014_705_MOESM4_ESM.pdf]

Supplementary Table 4

Confidence Intervals on the Baseline Inversion and Thatcherisation Effects for PHD

	Baseline Difference			Confidence Interval	
Variable	*M*	*SE*	Component Difference	Lower Bound	Upper Bound
Inversion Effect					
N170	<0.01	0.01	-0.09	-0.03	0.03
P2	<0.01	0.02	0.47	-0.04	0.03
P3b	<0.01	0.02	-0.01	-0.03	0.03
Eye Thatcherisation Effect					
N170	<0.01	0.01	-0.07	-0.03	0.03

Note. Difference score represents inverted condition – upright condition for inversion effect and normal – Thatcherised for eye Thatcherisation effect.

For Review Only
