# Supplementary material for: The Role of Configurality in the Thatcher Illusion: An ERP Study
Source: Psychon Bull Rev. 2014 Aug 8;22(2):445–52. doi: 10.3758/s13423-014-0705-3 (PMC4365276; doi:10.3758/s13423-014-0705-3)
Supplement: Supplementary file 5 — (PDF 223 kb) [file 13423_2014_705_MOESM5_ESM.pdf]

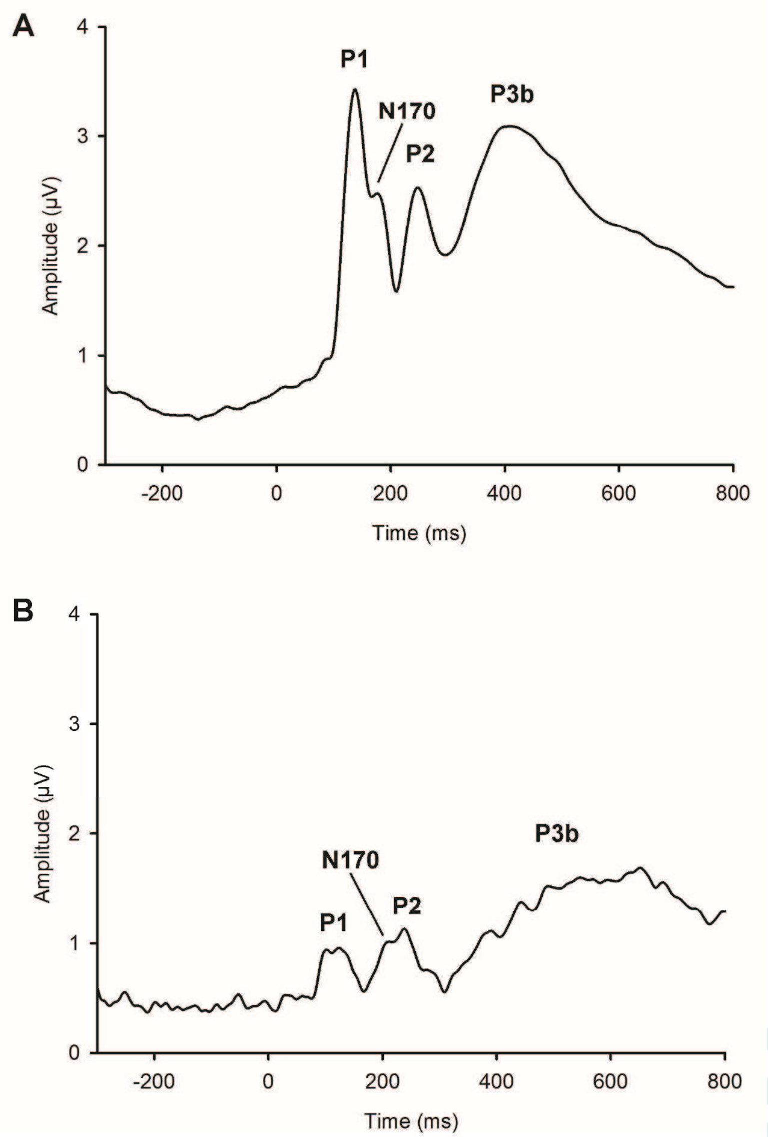

Supplementary Figure 1. Graph showing grand average global field power across the epoch for all electrodes, all conditions for (a) typical participants and (b) PHD. The graphs highlight the components P1, N170, P2 and P3b as the displayed peaks. The windows for peak detection were determined by the electrode of maximum amplitude at each component for typical participants. For the P1, I_z peaked at 138 ms, determining a window of 118 – 158ms; for the N170, TP_8 peaked at 176 ms, determining a window of 166-206 ms; for the N170, O_z peaked at 248 ms, determining a window of 166-206 ms; and for the P3b, CP_z peaked at 410 ms. determining a window of 318-518 ms.
