# Supplementary material for: The Role of Configurality in the Thatcher Illusion: An ERP Study
Source: Psychon Bull Rev. 2014 Aug 8;22(2):445–52. doi: 10.3758/s13423-014-0705-3 (PMC4365276; doi:10.3758/s13423-014-0705-3)
Supplement: Supplementary file 6 — (PDF 198 kb) [file 13423_2014_705_MOESM6_ESM.pdf]

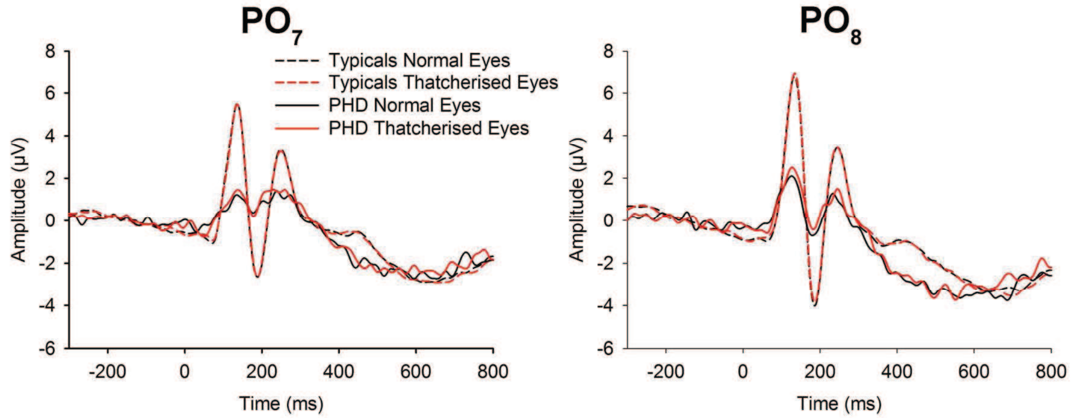

Supplementary Figure 2. Amplitude (µV) across normal and Thatcherised eye conditions at the (a) PO_7 and PO_8, electrodes providing an example of eye Thatcherisation effect for the right hemisphere N170 component for typical participants and PHD.

For Review Only
