# Supplementary material for: The Role of Configurality in the Thatcher Illusion: An ERP Study
Source: Psychon Bull Rev. 2014 Aug 8;22(2):445–52. doi: 10.3758/s13423-014-0705-3 (PMC4365276; doi:10.3758/s13423-014-0705-3)
Supplement: Supplementary file 7 — (PDF 145 kb) [file 13423_2014_705_MOESM7_ESM.pdf]

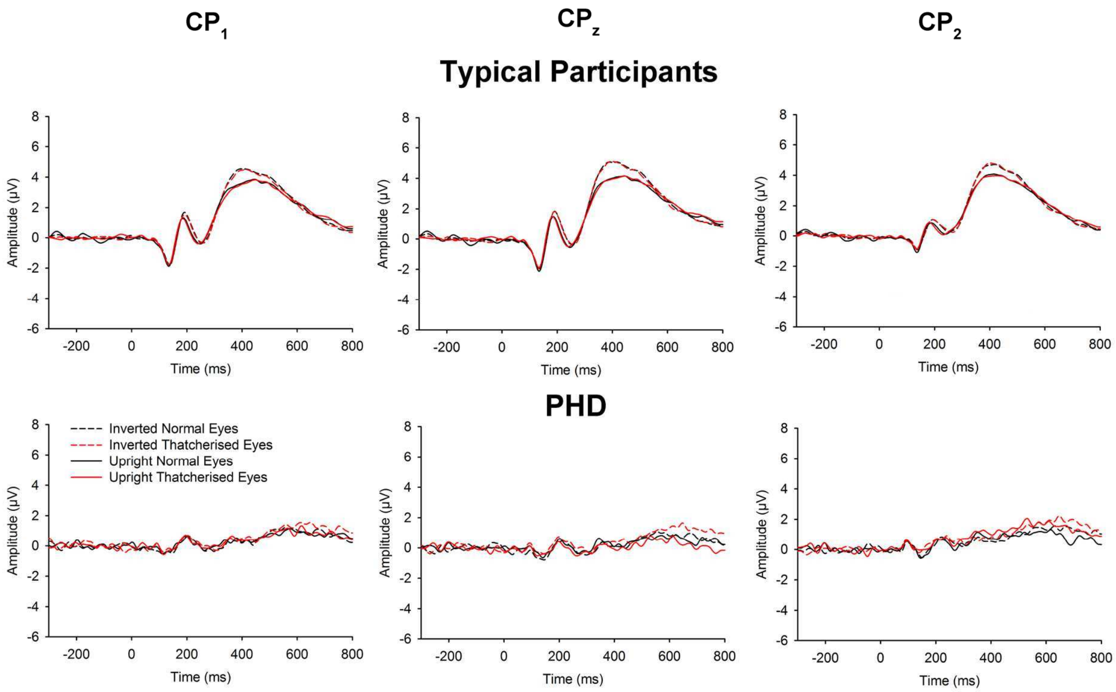

Supplementary Figure 3. Amplitude (µV) across normal and Thatcherised eye conditions at the CP_1, CP_z and CP_2 electrodes across orientation providing examples of eye Thatcherisation and orientation interaction at the P3b component for typical participants and not PHD.

Review Only
